# The Bottle of Medicine Habit

**Published:** 1923-04

**Authors:** 


### The Bottle of Medicine Habit.

SIR,—It is not only in the public houses where we should look for national income disappearing down the throat. I have had opportunities of studying Insurance Act prescriptions which lead me to think that a good deal of the money is wasted. Many of the prescriptions relate to drugs which are totally inactive when tested in the wards of a hospital; if they act at all it is only through the blessings of faith. Most of them cure by means of the even flowing flux of time.

Exception must be made for morphine and the coal-tar group of analgesics. These drugs certainly act, but as to the wisdom of prescribing them, opinions differ. Their use, in my view, is to be deprecated on every possible occasion, but it must be recognised that there is a vast body of well-considered opinion which holds to their use. The drugs which do something and have valuable, if dangerous, properties are not available under the National Insurance Act in what may be termed guaranteed quality. Till the last few months dispensing has not, I believe, been controlled, although the profession have been loud in their demands for proper control. The results of analyses have been very unsatisfactory. Dispensing has undoubtedly been bad, but some of the really crucial drugs are not susceptible of chemical analysis. Digitalis, the great heart drug, cannot be assayed by an ordinary analyst. Thyroid gland tablets are another example of the limits of chemical analysis.

You will be doing a public service if you insist that a vast amount of public money is spent in providing drugs which are useless either because the patient does not require drugs or because he is better without them.

A CONSULTANT.

